# The Potential Effects of Centipede Venom and Ethylenediaminetetraacetic Acid (EDTA) Leading to Pseudothrombocytopenia in an 11-Year-Old Girl

**DOI:** 10.7759/cureus.82822

**Published:** 2025-04-23

**Authors:** Nikita N Egbert, Umme Salma Rangwala, Andrew David, Vivek Sugadev, Alan V Koshy

**Affiliations:** 1 Pediatric Emergency Medicine, Christian Medical College, Vellore, IND; 2 Pediatrics, Dr. D. Y. Patil Medical College, Hospital and Research Center, Pune, IND; 3 Internal Medicine, Mahatma Gandhi Memorial Medical College, Indore, IND

**Keywords:** autoantibody, centipede bite, centipede venom, edta, edta-induced pseudothrombocytopenia, gpllb/llla, integrin, pediatrics, platelet cluster formation, pseudothrombocytopenia

## Abstract

Centipede bites are uncommon and mostly occur in subtropical regions. While most cases present with local swelling and tenderness around the bite site, with few systemic manifestations, the potential complications can be deadly. This case describes an unusual presentation of a centipede bite, in which the patient experienced an unexpected fluctuation in platelet counts, which occurred up to weeks after the bite. While the cause remains unclear, we question whether this occurred solely due to the direct effects of the centipede venom or if there were other factors at play.

The aim of this report is to explore the possible differentials. Most importantly, we shed light on a plausible but often overlooked cause of thrombocytopenia; ethylenediaminetetraacetic acid-induced pseudothrombocytopenia, the anticoagulant present in blood sample collection tubes. Similar cases have yet to be reported to our knowledge. Physicians must maintain a high degree of suspicion when facing unexplained thrombocytopenia in clinical practice.

## Introduction

The earliest fossils of centipedes are over 400 million years old. The natural process of evolution has allowed them to develop into very effective predators. While over 3,500 species are known, only 15 of these, i.e., about 0.5%, can cause symptoms in patients and, rarely, mortality [1].

Centipede bites have been reported in many tropical countries, such as South America, India, and Africa. Unsuspecting people are often bitten on the lower limbs at night in the hot and wet months of the year. There is a paucity of statistics regarding the occurrence of bites, likely due to underreporting of incidences. However, Mohanty et al. [2] reported that out of the 60 patients who visited the emergency department (ED) after being bitten by a centipede, only three patients experienced a sudden progression of local symptoms that warranted a repeat visit.

The effects of centipede venom are brought about by various enzymes, some yet to be understood by modern science. The well-studied ones, to name a few, are phospholipases, which disrupt the cell’s membrane integrity; metalloproteases, which damage the extracellular matrix protein; and hyaluronidases, which facilitate the spread of venom. Venom constituents also include various proteins without enzymatic activity, such as disintegrins, hemolysins, cardiotoxins, myotoxins, and neurotoxins [3]. Centipede venom is also known to have platelet-aggregating properties, the mechanism of which remains unclear. Several components of the venom are collectively responsible for symptoms such as extreme local pain and transient bleeding. Although recovery is spontaneous in most patients, fatal complications like secondary infections, acute myocardial infarcts, acute coronary ischemia, acute renal damage, or anaphylaxis are known to occur [4].

Since no antivenom exists, management of centipede bites is often limited to supportive treatments like wound care, ice fomentation, and pain control with the use of oral antihistamines and anti-inflammatory medications. Additional measures can include a tetanus booster, topical anesthetics, and antibiotics to prevent secondary bacterial infections [3,4]. Ethylenediaminetetraacetic acid (EDTA) is a common anticoagulant present in blood sample collection tubes. It prevents blood clot formation by chelating calcium ions in the blood sample. A lesser known consequence of anticoagulation with EDTA is a phenomenon known as EDTA-induced pseudothrombocytopenia (PTCP). It is caused by in vitro platelet clumping, leading to a falsely low platelet count (PC). In the general population, the incidence of EDTA-induced PTCP is 0.03%-0.27% [5].

Although the exact mechanism of this EDTA-induced PTCP is yet to be discovered, some studies have suggested an immunological basis for this phenomenon. The platelet surface glycoprotein (Gp), GpIIb/IIIa, is a calcium-dependent heterodimer. Chelation of calcium by EDTA causes the dissociation of this heterodimer, exposing certain GpIIb/IIIa antigens that are then recognized and attacked by autoantibodies targeting those specific antigen epitopes. This leads to platelet clumping and the automated analyzer machines reading low PC [5,6].

We report a unique case of a centipede bite in a pediatric patient, in which a variation in PC was observed up to 20 days after discharge. An interesting differential highlighted in this review is the potential for the undulating PC to be due to EDTA-induced PTCP. An abstract scientific consideration we have discussed in detail is that the PTCP could be linked to inflammation and, possibly, autoantibody formation, resulting from the effects of the centipede venom. To the best of our knowledge, a similar case has not been reported before.

## Case presentation

The 11-year-old girl came to the pediatrics ED with a complaint of a centipede bite, which occurred at 2 p.m., two hours before arrival at the hospital. Her mother reported that a centipede had been seen crawling near the area. The patient was bitten once on the dorsal aspect of the right foot, proximal to the fourth webbed space.

On examination, there was a small bite wound with surrounding redness, pain, and swelling that extended to the ankle. There was no restriction to the range of motion at the ankle joint; distal pulses were palpable. The patient experienced two episodes of vomiting following the bite. There were no other systemic symptoms, discharge, or bleeding manifestations from the bite site.

Initial management at a primary care center consisted of a tetanus toxoid injection and dexamethasone. The patient was then referred to our tertiary care center. In the ED, vital signs are as follows: heart rate of 123/minute, blood pressure of 128/84 mmHg, respiratory rate of 26/minute, and a temperature of 98.2°F. The right lower limb was elevated due to the swelling, and the pain was managed with intravenous paracetamol and tramadol. Additional medications administered included prazosin (an alpha-1 adrenergic receptor inhibitor, which allows for vasodilation, used in scorpion stings to prevent fatal shock), ondansetron, and vitamin K.

Laboratory investigations, such as serum electrolytes, creatinine, and coagulation studies, were all within normal limits.* *Table 1 lists laboratory values of tests done in our ED. However, the complete blood count (CBC) revealed a PC of 13,000/mm^3^ and a white blood cell count (WBC) of 21,000/mm^3^ with a neutrophil predominance of 79%. The increased WBC count was attributed to physiological stress or dexamethasone given earlier.

**Table 1:** Laboratory values at the time of arrival to the hospital WBC: white blood cells; ALT: alanine transaminase; AST: aspartate transaminase; ESR: erythrocyte sedimentation rate; PT: prothrombin time; INR: international normalized ratio; APTT: activated partial thromboplastin time; SGPT: serum glutamate pyruvate transaminase; SGOT: serum glutamate oxaloacetate transaminase; CK-MB: creatine kinase-myocardial band

Parameter	Value	Units	Reference range
Hemoglobin	12.4	g/dL	M: 13-17, F: 12-16
Platelet count	13,000	/mm^3^	150,000-450,000
WBC total	21,000	/mm^3^	4,000-12,000
Neutrophils	79	%	-
Eosinophils	1	%	-
Lymphocytes	18	%	-
Basophils	0	%	-
Monocytes	2	%	-
PT	13.3	Seconds	11.7-16.1
INR	0.99	-	-
APTT	25.4	Seconds	22-34
ALT (SGPT)	11	U/L	7-56
AST (SGOT)	20	U/L	10-40
Creatinine	0.59	mg/dL	0.5-1.4
Sodium (Na)	143	mmol/L	135-146
Potassium (K)	3.6	mmol/L	3.5-5.0
D-dimer	290	ng/mL	150-450
Fibrinogen	403	mg/dL	-
Troponin-T	5.9	pg/mL	Up to 14
CK-MB mass	2.5	ng/mL	M: 0-6.22, F: 0-4.88

Further history did not reveal any personal or family history suggestive of hematologic or bleeding disorders. The patient had a history of an upper respiratory tract infection one month prior, at which time CBC parameters were within normal limits, supporting the absence of any bleeding diathesis.

The patient was transferred to the pediatric intensive care unit (ICU) because of low PC, in anticipation of the possible requirement of a blood product transfusion, as well as for close monitoring and further management. A surgery consultation was requested due to the progression of the swelling, which had extended beyond the ankle and up to the knee. The patient was started on antibiotics for possible cellulitis.

**Figure 1:**
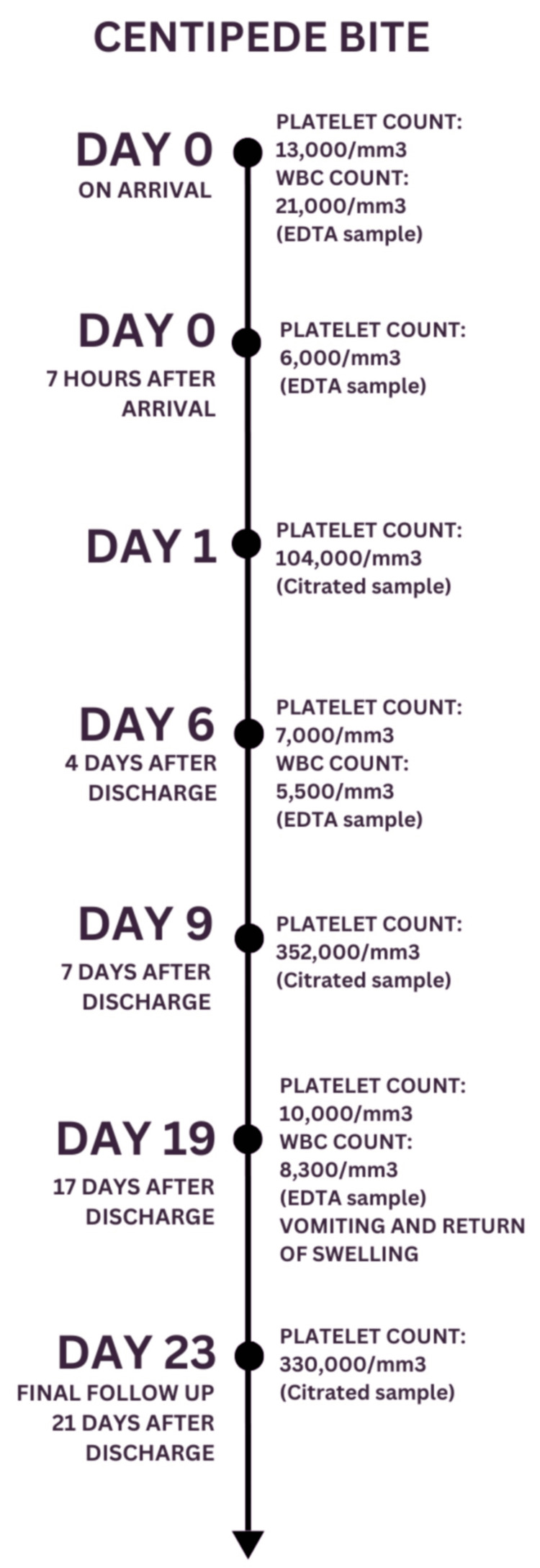
Timeline of hospital stay and follow-up period demonstrating waxing and waning of PC WBC: blood cell count; EDTA: ethylenediaminetetraacetic acid; PC: platelet count

Tests for troponin-T, creatine kinase myocardial band, fibrinogen, and D-dimer were within normal limits. Figure 1 presents a timeline illustrating the evolution of PC trends. A repeat PC done seven hours after hospital admission revealed a level of 6,000/mm^3^. A peripheral smear showed a count of <15,000/mm^3^ with clustered platelets. At this point, the pathology laboratory alerted the physician to the possibility of EDTA-induced PTCP, and a sample using citrate as an anticoagulant was requested. The result was a PC of 104,000/mm^3^.

The patient did not develop any complications such as compartment syndrome, rhabdomyolysis, myocardial infarction, or anaphylaxis. She was subsequently discharged with instructions for follow-up.

At the first follow-up visit, on day 6 after the bite, the EDTA sample showed a PC of 7,000/mm^3^ and a WBC count of 5,500/mm^3^. There were no symptoms associated with thrombocytopenia. On day 9, a citrated sample revealed a PC of 352,000/mm^3^. By this time, the swelling of the right lower limb had resolved.

On day 19 after the bite, the PC was again found to be 10,000/mm^3^ on an EDTA sample, with platelet clusters noted on the peripheral smear. The patient’s parents reported that swelling and redness of the right lower limb had reoccurred four days earlier, accompanied by a single episode of vomiting. To rule out deep vein thrombosis, a Doppler study was performed, which showed evidence of active inflammation (increased vascularity and tissue edema), likely indicative of cellulitis. The patient was prescribed amoxicillin and clavulanic acid.

At the final follow-up appointment, 23 days after the bite, the patient's PC was 330,000/mm³, which was within the normal physiological range. There were no local signs or symptoms at the site of the centipede bite. The timeline of hospital stay and follow-up period demonstrates the waxing and waning of PCs.

## Discussion

Centipedes, their venom, and bite management

**Figure 2:**
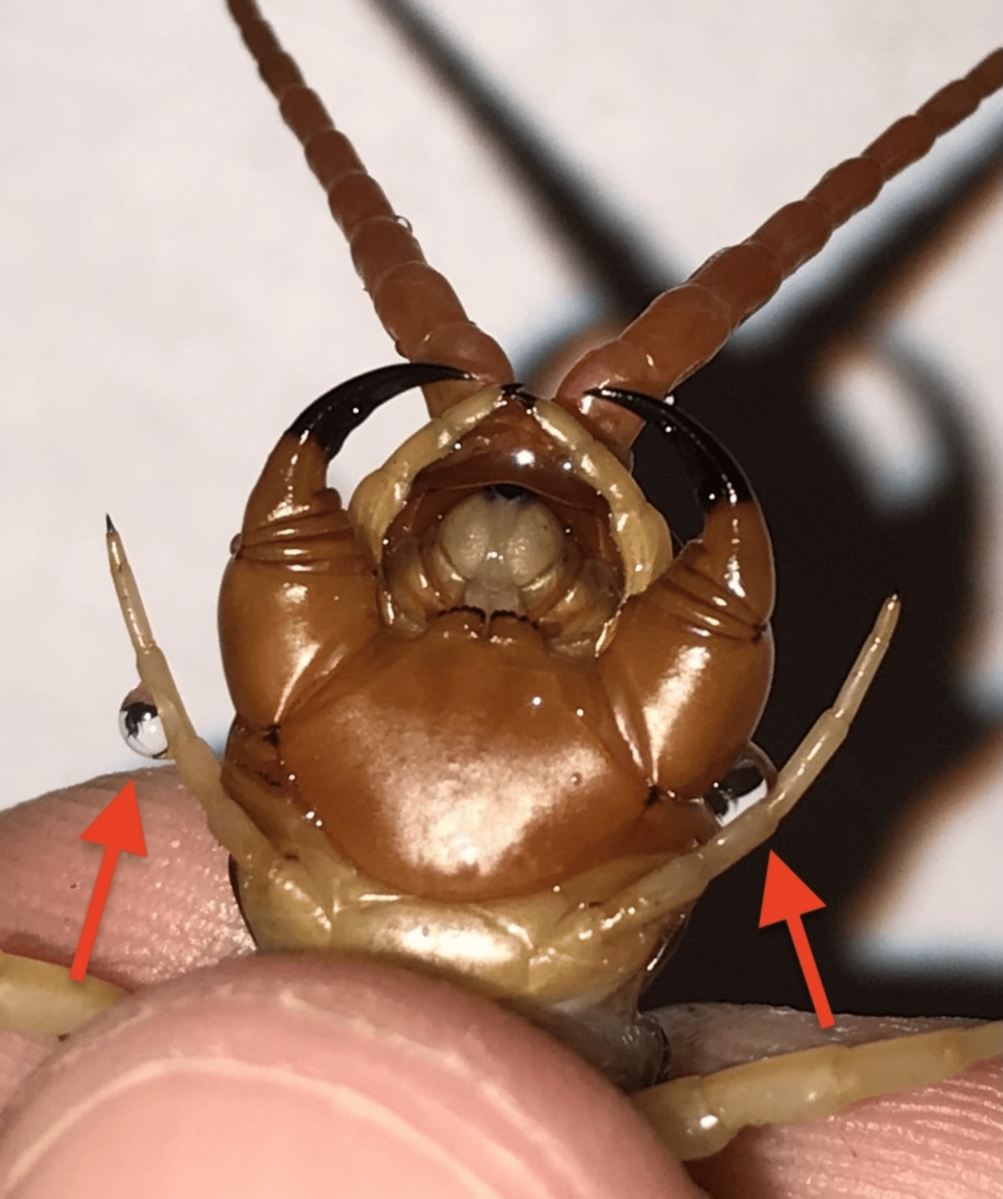
Forcipules with drops of venom (red arrows) Source: Reused with permission from the owner of the image [8]

Centipedes are from the phylum Arthropoda and are further subdivided into the class Chilopoda. Most centipede bites inflicted on humans are from the Scolopendra family. These carnivorous predators are the largest centipedes currently identified. They can grow up to 12 inches in length and have anywhere from 15 to 200 segments. Each segment has one pair of legs, along with two long, claw-like structures, called forcipules, that originate from the first pair of legs. Forcipules, used to inject venom from glands for defense or to catch prey, are shown in Figure 2. The larger size of some centipedes likely reflects the amount of venom available for injection [1,7].

Unlike other venomous animals, much is yet to be known about the mechanism of action of centipede venom. Approximately 50 components of centipede venom have been identified. In larger doses, it can cause myotoxic, cardiotoxic, and neurotoxic effects as well as other systemic symptoms. Venom components are extremely complex and include peptide toxins that contain disulfide bonds (which increase the stability of the venom), which exert a neurotoxic effect by causing local pain and paralysis. A component called polymodal transient receptor potential vanilloid 1 has also been identified. This is a capsaicin receptor that responds to heat and causes excruciating pain [9]. Centipede venom is also rich in natural bioactive proteins, peptides, and multiple other small molecules such as amines, serotonin, polysaccharides, and lipids [9,10]. For this reason, they have been used for centuries as traditional medicines to treat conditions like stroke, epilepsy, tetanus, burns, tuberculosis, inflammation, arthritis, and tumors. Thus, extensive research is being conducted to explore the possible modern therapeutic uses [9].

Centipede bites can be identified by double fang marks. They cause an intense, burning type of pain, itching, erythema, and local edema. Some patients may also have headaches, malaise, anxiety, dizziness, chills, fever, and weakness. Most victims recover spontaneously; however, the most common complication of bites is secondary bacterial infection leading to cellulitis, lymphedema, and lymphangitis. Secondary infections and anaphylaxis are the most common causes of mortality. In rare cases, severe local manifestations such as superficial necrosis may occur [4,7,9]. Other rare systemic complications that physicians must be aware of include myocardial ischemia and infarction, thrombocytopenia, rhabdomyolysis, and renal injury causing hematuria and hemoglobinuria [9]. A study by Harada et al. [11], in Japan, showed cross reactivity between bee stings and centipede bites, indicating that patients who are prone to developing severe reactions to bee stings have a higher chance of developing an anaphylaxis-like reaction from a centipede bite.

While there are currently no guidelines for managing a centipede bite, there is an outline of supportive care. Initial measures include washing the site of the bite with soap and water, applying ice packs for prehospital management, using hot water immersion, administering analgesics, and prescribing antihistamines. Currently, there is no antivenom available [12]. In the hospital, the patient benefits from early wound disinfection, inquiry of antitetanus vaccination status, and prophylactic antibiotics to prevent bacterial superinfection [13]. Local swelling may recur within three weeks after the bite, as was the case in our patient. Generally, the prognosis of a centipede bite is favorable, in that the symptoms usually last up to 48 hours and resolve spontaneously [14].

EDTA-induced PTCP

PTCP is defined as a falsely low platelet count. A few possible causes include abnormally large-sized platelets, the presence of cold agglutinins, numerous microclots, and incorrect blood sampling techniques [6].

While EDTA-induced PTCP is a rare phenomenon, one of the earliest studies known on the topic of PTCP was done in 1981 by Manthorpe et al. [15]. It noted that there was a variation of PC while using EDTA versus a citrate solution to stabilize blood samples. They also managed to demonstrate antibodies against platelets through experiments using an immunofluorescence technique. Thereafter, findings of several studies supported the hypothesis that an antibody-antigen reaction is responsible for PTCP.

The cell membrane of platelets incorporates several integrins, which are integral to the structure and function of the cell. Some of these serve a functional role as receptors for intermediates of the coagulation cascade and cytoskeletal components. Vital integrins called Gp include GpIb/IIa (collagen receptor), GpIb/Va (fibronectin receptor), GpIb/VIa (laminin receptor), Gp IIIb/Va (vitronectin receptor), and, most relevant to this paper, Gp IIb/IIIa (fibrinogen receptor).

GpIIb/IIIa is the most prevalent integrin present both on the surface of platelets and intracellularly. It surfaces upon platelet activation and brings about platelet adhesion and thrombus formation. It does so by binding to ligands, which include fibrinogen, fibrin, von Willebrand factor, thrombospondin, fibronectin, and vitronectin [16]. A widely accepted hypothesis is that EDTA-induced PTCP is caused by antiplatelet antibodies against Gp [17]. IgM, IgA, and, most commonly, IgG antibody subtypes are implicated in this phenomenon [5].

EDTA-induced PTCP has been associated with a large variety of conditions. These most commonly include viral infections, including COVID-19, sepsis, liver diseases, autoimmune conditions, malignancy, thrombotic disorders, and cardiovascular disorders. Upon receiving a machine alert for dangerously low PC in the absence of clinical signs, a peripheral smear should be done for further assessment. Since EDTA-induced PTCP is a temperature and time-sensitive phenomenon, measures to minimize it include maintaining an optimum temperature of 37°C before testing and promptly analyzing the sample, since PC can fall as early as one minute after sample collection [5,18]. A step-up protocol for the evaluation of thrombocytopenia using alternative anticoagulants has been described; blood is first collected in EDTA (step 1), then sodium citrate (step 2), lithium heparin (step 3), sodium fluoride (step 4), and, finally, ammonium oxalate (step 5), which is evaluated in a Neubauer chamber for the presence of platelet clumps at each step [19].

Effects of centipede venom may set the stage for EDTA-induced PTCP

**Figure 3:**
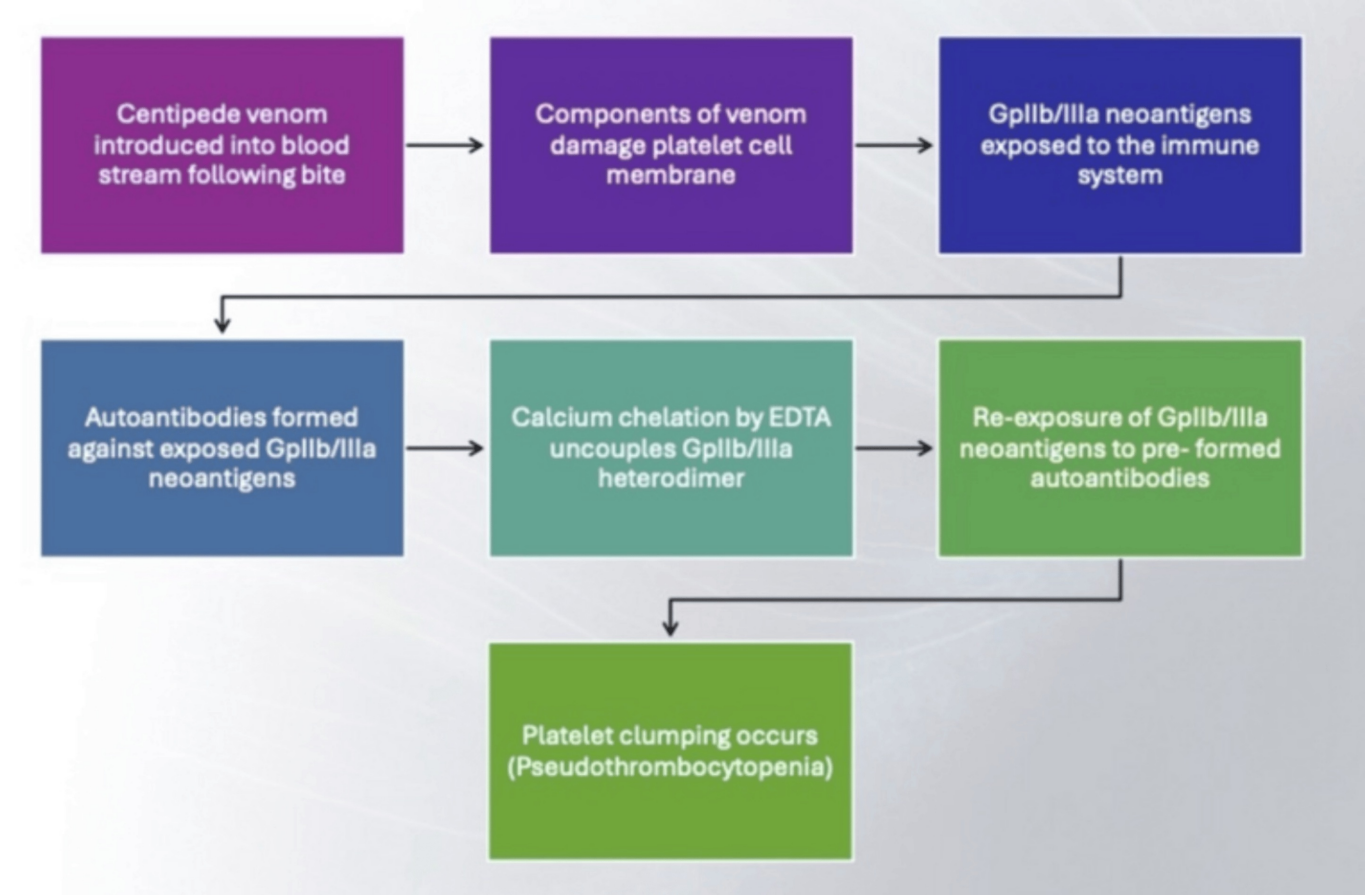
Overview of correlation between centipede venom and EDTA-induced PTCP EDTA: ethylenediaminetetraacetic acid; PTCP: pseudothrombocytopenia

Considering the immunological basis of PTCP, i.e., autoantibodies reacting to platelet antigens, the events that likely led to this in our patient have been summarized in Figure 3 and described as follows. A trigger, such as centipede venom, leads to autoantibody formation. This occurs due to constituents such as phospholipases and metalloproteases (in the venom) that damage and destabilize the cell membrane of platelets, causing extrusion of their intracellular contents.

Most relevant to this case report are the peptide components of centipede venom, especially disintegrin. Once the platelet membrane damage occurs, disintegrin breaks down the integrin GpIIb/IIIa, resulting in the exposure of neoantigens. The immune system identifies these neoantigens as foreign and develops autoantibodies against them [17].

The mechanism by which EDTA prevents blood samples from coagulating is calcium ion chelation. This chelation is responsible for the uncoupling of the GpIIb/IIIa heterodimer [6], effectively causing the reexposure of GpIIb/IIIa neoantigens, against which autoantibodies were already formed by the centipede venom insult. The preformed autoantibodies attack GpIIb/IIIa neoantigens, resulting in the platelet clumping and PTCP.

The absence of true thrombocytopenia in our patient was substantiated by the normal PCs on citrated samples as well as the lack of signs of active bleeding and a negative history of any bleeding diathesis. This suggests that the fluctuating PC was primarily EDTA-induced, perhaps aggravated by the lasting effects of the venom from the centipede bite.

An age-old question is whether PTCP can be passed from a donor to a recipient through blood product transfusions. While, in general, blood donation is avoided during flare-ups of autoimmune conditions, there has been no evidence to suggest that there should be concern when dealing with donors with a history of PTCP. However, it has been noted that transplacental transmission of PTCP can occur in utero, further proving an antibody component [19].

The action of disintegrin has been exploited to create medications. A well-known drug, eptifibatide, has been derived from barbourin, a snake venom disintegrin. While its action is not that of a disintegrin, its similar structure allows it to bind to Gpllb/llla integrins, thereby hindering the coagulation cascade, preventing platelet aggregation and clot formation [16].

## Conclusions

This review investigates the occurrence of EDTA-induced PTCP and its association with the effects of centipede venom. The inconsistency in the PCs obtained from the EDTA vs. citrated samples in this patient compelled us to take into consideration EDTA-induced PTCP. While statistics show that this is a rare phenomenon, there is likely underreporting due to a lack of awareness, making it impossible to estimate the true incidence.

This paper aims to expand the knowledge of the common doctor. The fact that the most commonly available anticoagulant in blood collection bulbs, EDTA, can cause PTCP is absolutely astounding. This has widespread global implications; it not only leads to the incorrect allocation and overutilization of precious medical resources but also results in painful and risky interventions for patients. On a more sobering note, physicians may opt to withhold essential treatments, like in the case of hematological malignancies, based on the assumption that low PCs are due to chemotherapy side effects.

The major concern lies in hospital departments that care for fragile patients, including neonatal ICUs, pediatric ICUs, adult ICUs, and EDs, which require effective decision-making in a timely manner, at the risk of losing a patient. Preparation of interventions like blood transfusions is often done before confirmation on repeat testing due to the emergent nature of thrombocytopenia. Confirmatory testing using alternative anticoagulants must be prioritized before medical interventions.

In this case, the physicians relied on the clinical presentation of our patient. The major decision of blood product transfusion was deferred based on the lack of concerning clinical features. This prevented a big impact decision from being taken solely based on laboratory values.

Technology has undoubtedly advanced the cause of patients in the practice of medicine. While modern medicine has instilled in physicians a heavy reliance on laboratory values and a timely report of counts, the awareness of EDTA-induced PTCP can help physicians minimize wasteful interventions by focusing on signs and symptoms, thereby putting the patient, as an individual, at the forefront of their decisions.
